# Born Too Soon: Care during pregnancy and childbirth to reduce preterm deliveries and improve health outcomes of the preterm baby

**DOI:** 10.1186/1742-4755-10-S1-S4

**Published:** 2013-11-25

**Authors:** Jennifer Requejo, Mario Merialdi, Fernando Althabe, Matthais Keller, Joanne Katz, Ramkumar Menon

**Affiliations:** 1 Partnership for Maternal, Newborn & Child Health, Geneva, Switzerland; 2 World Health Organization, Geneva, Switzerland; 3 Institute for Clinical Effectiveness and Health Policy, Buenos Aires, Argentina; 4 University Hospital Essen, Essen, Germany; 5 Johns Hopkins Bloomberg School of Public Health, Baltimore, USA; 6 The University of Texas Medical Branch at Galveston, Galveston, USA

## Abstract

**Abstract:**

Pregnancy and childbirth represent a critical time period when a woman can be reached through a variety of mechanisms with interventions aimed at reducing her risk of a preterm birth and improving her health and the health of her unborn baby. These mechanisms include the range of services delivered during antenatal care for all pregnant women and women at high risk of preterm birth, services provided to manage preterm labour, and workplace, professional and other supportive policies that promote safe motherhood and universal access to care before, during and after pregnancy. The aim of this paper is to present the latest information about available interventions that can be delivered during pregnancy to reduce preterm birth rates and improve the health outcomes of the premature baby, and to identify data gaps. The paper also focuses on promising avenues of research on the pregnancy period that will contribute to a better understanding of the causes of preterm birth and ability to design interventions at the policy, health care system and community levels. At minimum, countries need to ensure equitable access to comprehensive antenatal care, quality childbirth services and emergency obstetric care. Antenatal care services should include screening for and management of women at high risk of preterm birth, screening for and treatment of infections, and nutritional support and counselling. Health workers need to be trained and equipped to provide effective and timely clinical management of women in preterm labour to improve the survival chances of the preterm baby. Implementation strategies must be developed to increase the uptake by providers of proven interventions such as antenatal corticosteroids and to reduce harmful practices such as non-medically indicated inductions of labour and caesarean births before 39 weeks of gestation. Behavioural and community-based interventions that can lead to reductions in smoking and violence against women need to be implemented in conjunction with antenatal care models that promote women's empowerment as a strategy for reducing preterm delivery. The global community needs to support more discovery research on normal and abnormal pregnancies to facilitate the development of preventive interventions for universal application. As new evidence is generated, resources need to be allocated to its translation into new and better screening and diagnostic tools, and other interventions aimed at saving maternal and newborn lives that can be brought to scale in all countries.

**Declaration:**

This article is part of a supplement jointly funded by Save the Children's Saving Newborn Lives programme through a grant from The Bill & Melinda Gates Foundation and March of Dimes Foundation and published in collaboration with the Partnership for Maternal, Newborn and Child Health and the World Health Organization (WHO). The original article was published in PDF format in the WHO Report "Born Too Soon: the global action report on preterm birth" (ISBN 978 92 4 150343 30), which involved collaboration from more than 50 organizations. The article has been reformatted for journal publication and has undergone peer review according to *Reproductive Health*'s standard process for supplements and may feature some variations in content when compared to the original report. This co-publication makes the article available to the community in a full-text format.

## The pregnancy period and childbirth - a critical window of opportunity

Pregnancy and childbirth represent a critical window of opportunity for providing effective interventions to prevent preterm birth and other adverse health outcomes associated with an early birth. These interventions encompass services delivered during antenatal care for all pregnant women and women at high risk of preterm birth, services provided to manage preterm labour, and interventions targeted at improving health behaviours and knowledge about early warning signs of pregnancy complications, including preterm labour. They also include the provision of needed social and financial support to disadvantaged mothers as well as workplace, professional and other supportive policies promoting safe motherhood and women's universal access to care before, during, and after pregnancy.

This paper is based on chapter four in the Born Too Soon Global Action Report on Preterm Birth launched in May, 2012 [1] and is part of a series entitled "Born Too Soon". The aim of the paper is to provide a summary of latest information about available interventions that can be delivered during pregnancy to reduce preterm birth rates and improve the health outcomes of the premature baby. A second objective is to describe promising avenues of research on the pregnancy period that will contribute to a better understanding of the causes of preterm birth and ability to design interventions at the policy, health care system and community levels for scale-up. An action agenda is proposed outlining opportunities for scaling up effective interventions in low- and middle-income countries and identifying priority research themes that will ultimately translate into the development of preventive and treatment interventions for universal application.

Exhaustive systematic reviews on preterm birth interventions for the pregnancy period serve as the source of information for the interventions or intervention areas described with proven or potential effectiveness in either reducing preterm birth rates or improving birth outcomes for the premature baby [2-11] including the Cochrane database (The Cochrane Collaboration, 2013), which includes regularly updated systematic reviews on available evidence from clinical trials, considered the most robust evidence for guiding policy and programme development. Additional articles used to support statements made throughout the paper were identified through PubMed and Google Scholar.

Blencowe and colleagues describe in detail the complex risk factors and multiple mechanisms believed responsible for the two types of preterm delivery, spontaneous and indicated, which includes possible epigenetic/genetic predispositions [12]. Advancements in the field of epigenetics have found links between fetal methylation of genes and the preterm birth pathway [13]. The majority of preterm births occur spontaneously, often following Preterm Premature Rupture of the Membranes (pPROM). Medically and non-medically indicated preterm births are related to early induction of labour or caesarean birth. Medical indications include pregnancy complications such as placental abnormalities (e.g., placenta previa), multiple gestations, maternal diabetes, pre-eclampsia and pre-existing conditions such as high blood pressure, asthma, thyroid and heart disease. Strategies for preventing or managing spontaneous and indicated types of preterm birth during the pregnancy period may differ due to variances in their underlying pathophysiological processes. Similarly, and for the same reason, clinical management of pre-existing conditions that may be exacerbated by pregnancy may differ from strategies used to address complications that develop during the pregnancy period. There are a large number of preventive interventions for spontaneous and indicated types of preterm birth that are currently part of the standard of care in high-income countries. Many of these interventions are not readily available or feasible to introduce in low- and middle-income countries, and supportive evidence of their effectiveness is lacking [14].

Available and proposed preterm birth interventions are tailored for delivery to three population groups: (1) all pregnant women; (2) pregnant women with a history of previous preterm delivery or other risk factors, including multiple gestation, bleeding during pregnancy, hypertensive disorders and diabetes; and (3) pregnant women experiencing or recovering from preterm labour. Interventions for the first two population groups are aimed primarily at prevention and range from basic and specialized packages of antenatal care services as well as supportive workplace and professional policies. Interventions designed for the third group are focused on improving the health outcomes of the premature baby. At the individual level, quality clinical management involves practitioners identifying each woman's own set of risk factors throughout the pregnancy and childbirth periods and responding with appropriate care.

This paper is divided into several sections. The first section describes services that can be provided during antenatal care visits to all women and to women at high risk of preterm delivery, and services that can be delivered to women experiencing preterm labour. The next section presents information on interventions that can be provided at the community level and in the policy arena to prevent preterm birth and improve obstetric outcomes for women and their babies. Table 1 summarizes the supportive policy and community measures that can be introduced, and the type of evidence-based services that can be provided during antenatal care and when a woman is experiencing preterm labour.

**Table 1:** Priority evidence-based interventions during pregnancy to reduce preterm birth rates and to benefit the premature baby

**Services delivered during antenatal care:**
• Basic package for all pregnant women
• Situational interventions for populations of women at high exposure risk (e.g. identification and treatment of malaria, tuberculosis and HIV)
• Behavioural, social support and financial interventions for disadvantaged women
**Management of pregnant women at higher risk of preterm birth including:**
• Identification and treatment of pre-existing conditions (e.g., diabetes, thyroid disease, heart disease, asthma and other chronic conditions)
• Identification and treatment of pregnancy complications (e.g., pre-eclampsia, antepartum haemorrhage)
• Monitoring multiple pregnancies
• Administration of progesterone to prolong pregnancy
• Identification and treatment of structural abnormalities (e.g., cervical cerclage, cervical pessary)
**Management of women in preterm labour including:**
• Tocolytics to slow down labour
• Antenatal corticosteroids to reduce mortality in the newborn
• Antibiotics for pPROM to prevent infection
• Provision of magnesium sulphate for neuro-protection of the newborn
**Community interventions:**
• Promote antenatal and skilled birth care for all women
• Smoking cessation programmes
• Reductions in exposure to secondhand smoke and other pollutants
**Policy interventions:**
• Policies to support safe motherhood and universal access to antenatal care
• Workplace policies regulating working hours and strenuous working conditions
• Professional and hospital policies to regulate infertility treatments and to reduce caesarean section rates and early induction of labour

Source: Born Too Soon report [1].

Following a short section on data limitations, the last section explores an action agenda, describing opportunities for scaling up programmes, identifying priority research themes, and listing a set of steps countries can introduce now to address the growing problem of preterm birth.

## Section I. Delivering effective care to women during pregnancy and preterm labour

Increasing access to care during pregnancy for all women is an essential step towards addressing the growing problem of preterm birth. Research has shown that women who receive antenatal care services are at lower risk for having a preterm birth than women who are not reached by the health system prior to delivery [15]. Further studies are needed to determine if this association is related primarily to the timing, number, and content of services provided during antenatal care visits, or to differences between women who do and do not receive antenatal care [16,17].

Many countries around the world report high coverage levels of antenatal care, making antenatal care visits an opportune time to deliver proven interventions to all pregnant women) [18]. Yet, there are large and unacceptable inequities in coverage between and within counties that need to be addressed. It is critical that all pregnant women receive at least the basic package of recommended antenatal care services for their own health and the health of their unborn babies. This section describes services that can be provided during antenatal care to all women, and to women at high risk of a preterm birth. It also describes effective services that can be provided to women experiencing preterm labour to improve the survival chances of the newborn.

### Antenatal care services for prevention of preterm birth for all women

Effective and culturally appropriate care during pregnancy is essential for increasing the likelihood of positive birth outcomes [19]. Antenatal care is a service delivery platform through which all women can be reached at multiple times during pregnancy with a package of interventions that can prolong a healthy pregnancy and improve maternal and perinatal health. Basic services that can be delivered during antenatal care with a potential impact on reducing preterm birth rates include identification of women at high risk of pretermbirth; screening for and treatment of sexually transmitted diseases including HIV and other infections (tuberculosis, malaria, bacterial vaginosis, bacteriuria); identification and correction of malnutrition and nutrition counselling including on multiple micro-nutrient supplementation; counselling on birth preparedness and complication readiness for identification of early labour and other risk factors; and behavioural and social support interventions such as smoking cessation programmes and programmes aimed at the prevention of violence against women [11,19-25].

Infections during pregnancy can cross into the intra-amniotic cavity, establish intra-amniotic infection and result in preterm labour. In some women, such infections are associated with premature rupture of the membranes [6]. Screening for and treatment of infections such as asymptomatic bacteriuria and bacterial vaginosis during antenatal care may reduce preterm births, although study findings show inconsistent results. Systematic reviews and meta-analyses show that antibiotic treatment for infection and periodontal care does not reliably reduce the risk of preterm birth, and effectiveness may depend upon when during pregnancy the infection or periodontal disease is detected and treated. Three hypotheses have been proposed to explain the inconsistent findings of the effect of antibiotic treatment on reducing preterm delivery: 1) infections may be polymicrobial in nature and even broad spectrum antibiotics may not be sufficient to treat them all, 2) infections may not be the primary cause of preterm labour, and underlying [21,22] immunosuppressive conditions may be responsible for inducing alternate biomolecular pathways leading to preterm labour. Therefore, treating the bacterial infection alone may not be sufficient, and 3) clinical signs of infection based on immune biomarkers are not true indicators of infection due to heterogeneity in inflammatory markers of various intra amniotic pathogens. More research is needed on the inter-relationships between infection, pregnant women's immune response, and the cascade of events resulting in preterm birth [26]. Such research can inform the development of optimal screening and treatment protocols based on underlying risk factors that will take into consideration when in pregnancy, or prior to pregnancy, screening and treatment for infections should occur to reduce the preterm birth rate [15].

The advantages of prophylactic treatment for malaria (intermittent presumptive treatment during pregnancy for malaria [IPTp]) and the use of bednets to prevent malaria transmission in malaria-endemic areas on reducing preterm birth similarly needs further investigation. More rigorous studies examining the impact of HIV infection and the use of antiretrovirals and TB medication during pregnancy on the risk of preterm birth that control for disease stage and other potential confounding factors are also needed [2].

Women's nutritional status during pregnancy has been linked to preterm birth, with underweight and obese pregnant women at elevated risk of preterm birth and other poor obstetrical outcomes [6,27]. Malnutrition increases vulnerability to infection and predisposes pregnant women to adverse obstetrical outcomes including preterm delivery, although findings on obesity and the risk of preterm birth are mixed. Recent research suggests that the association between body mass index (BMI) and risk of preterm birth may differ by race or ethnic group, underscoring the complexity of the role nutrition plays in triggering preterm birth [28]. Providers can administer nutritional supplements and counselling services during routine antenatal visits. Evidence from clinical trials to date does not, in general, show a clear beneficial effect of nutritional counselling, or protein and calorie supplements during pregnancy on the preterm birth rate. A small effect on reducing preterm births has been found from the use of multiple micro-nutrient supplementation, and evidence suggests that calcium supplementation during pregnancy can reduce the incidence of preterm births in populations at risk of low calcium uptake [11]. New findings also suggest a link between supplementation of docosahexaenoic acid (DHA) and overall greater gestation duration and infant size [29]. Further research is needed to determine the optimal timing during a woman's life course of introducing nutritional interventions for reducing the risk of preterm birth and how best to implement these interventions within the broader context of efforts to improve the overall nutritional status of pregnant women and women of reproductive age. Pregnant women should be encouraged to take multi-vitamin supplements and to gain an adequate amount of weight based on their nutritional status at the beginning of their pregnancy, and for other health-promoting reasons such as reducing the risk of small for gestational age babies and neural tube defects.

### Antenatal care services for prevention of preterm births, women at higher risk

Women at increased risk of preterm delivery can be identified during antenatal care based on obstetric history (e.g., known uterine or cervical anomaly or previous preterm birth, pre-existing conditions such as chronic diseases) or presenting pregnancy characteristics (e.g., hypertensive disorder of pregnancy, diabetes, multiple gestation, bleeding). Young adolescents also are at greater risk [30,31]. Although prospective studies that evaluate the use of risk-screening tools based on epidemiologic, demographic biomarkers and clinical indicators in routine antenatal care are still needed, there are several approaches for providing preventive care for these women. One such approach includes specialized antenatal clinics for women with evident risk of preterm birth that provide enhanced health education, and more rigorous monitoring and treatment of risk factors and pregnancy complications. The evidence is scanty on such approaches in reducing the risk of preterm birth, but trials were conducted prior to the introduction of new screening tests. Another approach involves adopting the principles of personalized medicine that includes tailoring interventions to match each individual woman's risk profile.

Administration of progesterone to prolong pregnancy in high-risk women with a history of previous preterm birth has been shown effective in preventing a recurrence of preterm birth in these women and in decreasing the prevalence of low birthweight [2]. A recent clinical trial, however, found no advantage to vaginal progesterone therapy at 200 mg or 400 mg doses in the prevention of preterm birth in twin pregnancies [32]. Recent guidelines and professional opinion recommend administering vaginal progesterone to women with singleton pregnancies and short cervical length to reduce preterm birth and perinatal morbidity and mortality [33]. Most studies to date on the use of progesterone have been conducted in high-income countries. There is a need for evidence generation on progesterone use in resource-constrained settings including how this intervention can be scaled up at national level and the feasibility of introducing universal cervical length measurement screening.

Cochrane reviews have shown small reductions in preterm birth rates with treatment interventions for pre-eclampsia such as calcium supplementation. A recent randomized controlled trial has shown promising results for the use of a cervical pessary to lower rates of spontaneous preterm birth among women with a short cervix [34]. Results from another randomized controlled trial did not find any reductions in poor perinatal outcomes from use of a cervical pessary for prevention of preterm birth in women with a multiple pregnancy [35]. Another promising intervention in reducing the risk of preterm birth that requires further investigation is the placement of circumferential stitches on a structurally weak cervix (cerclage) [2,3].

These interventions are only applicable to a small number of high-risk women, however, and the overall effect on general population rates of preterm birth is likely to be limited.

### Management of women in preterm labour to improve survival chances of the premature baby

Once preterm labour has commenced, there are interventions that can prolong pregnancy and improve health outcomes and survival for the premature baby. To date, these interventions have not been designed to address the underlying mechanisms that trigger preterm labour.

Interventions to prolong pregnancy include the provision of tocolytic agents that inhibit uterine contractions to suppress labour (e.g., oxytocin antagonists, betamimetics, calcium channel blockers, magnesium sulphate), and clinical practices to ensure the optimal timing of caesarean birth and labour induction for indicated preterm births [2,9,15]. The provision of tocolytics has been shown effective in slowing down labour, enabling the administration of antenatal corticosteroids and transfer of mother and baby to a higher-level facility where appropriate care may be available. Any use of strategies to prolong labour, including delaying caesarean birth, must be evaluated against the potential risk of continued exposure of mother and fetus to sub-optimal conditions that may result in harmful effects. Further research is needed on the short- and long-term health consequences for mother and baby from efforts to prevent preterm labour.

There are three key interventions that can be delivered during the pregnancy period with evidence of effectiveness in improving health outcomes in babies born prematurely, although none of these are effective in reducing the incidence of preterm births: antenatal corticosteroids, antibiotics for pPROM, and magnesium sulphate.

**Figure 1:**
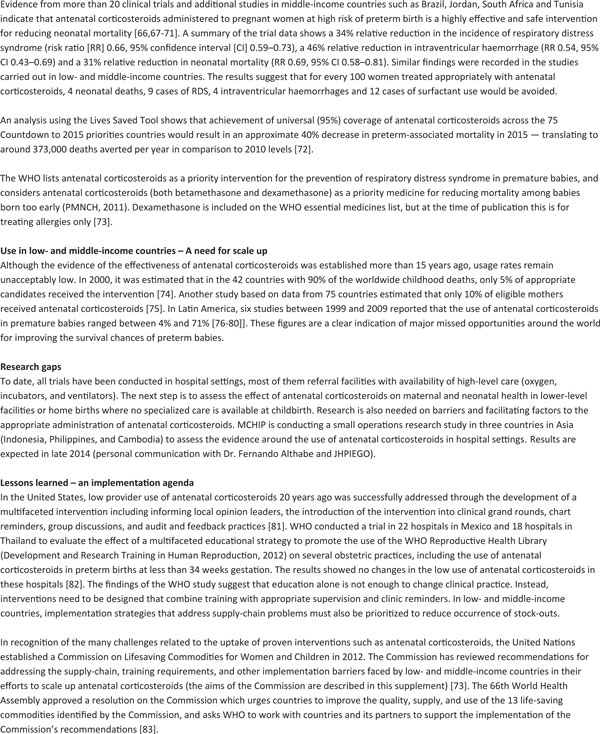
**Antenatal corticosteroids**. Source: Born Too Soon report [1].

The administration of antenatal corticosteroids to pregnant women at high risk of preterm birth possibly as early as 23 weeks can significantly reduce the premature baby's risk of death, respiratory distress and developmental problems (Figure 1) [8].

Preterm premature rupture of the membranes (pPROM) is strongly associated with infection of the amniotic membranes contributing to preterm birth and other poor fetal outcomes such as cerebral palsy and chronic lung disease. It is unclear if infection is a cause or consequence of pPROM. The incidence of pPROM has not measurably changed in the past 40 years, and there are currently no screening or diagnostic markers because the mechanisms responsible for a large proportion of pPROM cases are still poorly understood. Antibiotic treatment for pPROM has been shown to delay onset of labour for up to 48 hours and to reduce neonatal infections and abnormal cerebral ultrasound scans prior to hospital discharge [10]. Clinical management of pPROM including duration of administration of antibiotic prophylaxis depends upon the maternal-fetal condition, and other factors need to be considered such as timing of administration of antenatal corticosteroids and delivery.

The administration of magnesium sulphate to women at risk of preterm birth helps to protect the baby's brain, reduce rates of cerebral palsy and improve long-term neonatal health outcomes. Further studies are needed, however, to investigate side effects of the treatment for the mother (e.g., flushing, sweating, nausea, vomiting, headaches and a rapid heartbeat) and how these can be reduced [20].

## Section II. Avenues for intervening to improve the broader context

This section describes opportunities to intervene at the community and policy levels to ensure a supportive environment for pregnant women.

### Behaviour and community interventions for the prevention of preterm birth

Lifestyle factors including depression, intimate partner violence, smoking, substance abuse and stress are risk factors for preterm birth. Antenatal care models, including group models, that address these issues and provide social and financial support for pregnant women and particularly disadvantaged/at-risk population groups have been associated with reduced rates of preterm birth [36,37]. There are numerous efforts underway to determine how best to integrate psychological and behavioural interventions, including programmes to prevent violence, into antenatal care to improve preterm birth rates and other maternal and neonatal health outcomes. Controlled trials need to be designed to further test the efficacy of antenatal care approaches that incorporate social and financial support measures so that such approaches can be introduced at scale in settings where they are most needed. A recent systematic review and meta-analysis on the effects of women's groups practicing participatory learning and action showed that these strategies are a cost-effective approach to improving maternal and neonatal health outcomes [38]. More research is needed on how women's empowerment approaches can translate into reduced preterm birth rates.

Smoking during pregnancy is a well-known cause of preterm birth, especially preterm birth complicated by pPROM. Smoking cessation interventions in high-income countries that combine counselling with additional social support services have been found to significantly reduce preterm birth and should be adapted for application in low- and middle-income countries where the large majority of smokers live [39]. These approaches could prove to be more successful than large scale campaigns in changing women's smoking habits during pregnancy. Greater understanding of the biomolecular pathways through which the chemicals in cigarette smoke trigger deleterious events during pregnancy could potentially lead to new interventions targeted at smokers unable to quit when pregnant.

Other programmes such as the Healthy Babies are Worth the Wait™ campaign of the March of Dimes and the "humanization of childbirth" social movement in Brazil and other Latin American countries that educate women with healthy pregnancies about the advantages of vaginal delivery and waiting until 39 weeks to deliver should be promoted, particularly in contexts characterized by high and rising elective caesarean birth rates [40,41]. Health care provider education on the adverse health outcomes of late preterm birth and of inducing birth for non-medical reasons prior to 39 weeks has been shown to be effective in reducing preterm birth rates in some countries such as Brazil [40,42,43].

### Policy interventions to promote healthy pregnancies

Pregnant women can experience a reduced risk of preterm birth and other health benefits from professional and public policies based on sound scientific evidence. Blencowe and colleagues show that the risk of preterm birth increases in women with twins and higher-order births [12]. Policies on infertility treatments and use of assisted reproductive technologies directed to limiting the number of embryos that can be transferred have shown success in reducing the number of higher-order births and the associated high risk of preterm birth in Europe, Australia and the United States [15,44,45].

Hospital policies aimed at lowering the primary caesarean birth rate and early induction rates, particularly for non-medically indicated reasons, are part of a growing effort to prolong pregnancy, promote healthy newborn outcomes and reverse the increasing trend of preterm birth. Such policies are especially needed in regions where caesarean birth rates, and particularly elective caesarean birth rates, are high or rising like in Latin America and many high-income countries. Legislation mandating universal access to maternal health care services and eliminating user fees in low- and middle-income countries also have been shown to be an important prerequisite to ensuring all women receive antenatal care.

Workplace policies designed to promote healthy pregnancies and protect pregnant women from occupational hazards can potentially reduce the risk of preterm birth. Examples include, but are not limited to, time off for antenatal care visits, paid pregnancy leave for a set number of weeks and exemption from night shifts and tasks requiring heavy lifting or standing for long periods of time [46]. Studies have shown that carrying heavy workloads and working more than 5 days a week are associated with preterm birth [47]. Measures that can improve general working conditions are especially important for pregnant women in low- and middle-income countries where they are more likely to be engaged in agricultural labour and other physically demanding tasks.

Pregnant women can benefit from legislation reducing their exposure to potentially harmful environmental risk factors such as second-hand smoke and air pollution (combustion and household sources) [48]. There is growing interest in understanding and developing policy and programmatic solutions to the association between air pollution and adverse pregnancy outcomes including preterm birth [49-54]. For example, the UN Foundation houses the Global Alliance for Clean Cookstoves, a multi-partner effort launched in 2010 to promote clean and efficient cookstoves. Traditional cookstoves and open fires, the primary means of cooking and heating for nearly three billion people in the developing world, place pregnant women at increased risk of preterm birth and other poor obstetrical outcomes.

## Section III. Limitations of the evidence

The clinical trial literature shows a lack of evidence for many of the preventive interventions currently in use. The multi-causal and complex nature of preterm birth is likely responsible for single interventions not showing a significant public health effect and it is thus doubtful that rates of preterm birth can be reduced by the delivery of one single intervention. There is, therefore, an imperative need for well-designed studies examining the effectiveness of interventions delivered alone or as an integrated package of antenatal care. Epidemiological research is needed to assess the effectiveness of introducing a comprehensive set of science-based interventions delivered at the policy, health system and community or home levels on reducing the rate of preterm birth. The scientific literature and technical reports similarly show evidence for only a handful of interventions delivered during pregnancy that can improve the health outcomes of babies born too early, again stressing the need for more research on available and promising interventions. This emphasis on research and generation of quality data, however, needs to be balanced with the further promotion and worldwide scale up of interventions with proven effectiveness.

The brevity of the list of interventions delivered during the pregnancy period with evidence of effectiveness for preterm birth prevention and for improving survival chances of the premature baby is related to long-standing neglect of the newborn period and to insufficient research on the biological complexities of pregnancy and childbirth. The growing concentration of child deaths in the newborn period and the emergence of organizations and efforts focused on the newborn (e.g., The Healthy Newborn Partnership in the mid-2000s, and Saving Newborn Lives) have served as a catalyst for focused attention on preterm birth, a leading cause of newborn mortality [55,56]. There is an increasing emphasis on research that can inform the development of cohesive strategies for addressing the underlying determinants of preterm delivery with the goal of reducing the numbers of preterm births.

## Section IV. An action agenda and next steps

This section describes opportunities to scale-up available effective interventions, priority research themes to increase the evidence base on what works and how best to deliver proven interventions to reach all women with effective care, and a set of action points that can be adopted by countries now depending upon resource constraints.

### Program opportunities to scale up

Coverage of antenatal care (at least one visit) is approximately 80% worldwide, with coverage levels dropping to about 50% for four or more visits. Inequities in coverage are pervasive, with coverage levels of four or more antenatal care visits hovering around 40% for the least developed countries [18]. These figures indicate that efforts aimed at improving availability of antenatal care, demand for care and women's ability to reach these services throughout the pregnancy period are needed, particularly in low-resource settings and amongst the most disadvantaged population groups. Adolescents are another group at high risk of preterm birth due to their young age and typically limited access to preconception and prenatal care [57].

**Figure 2:**
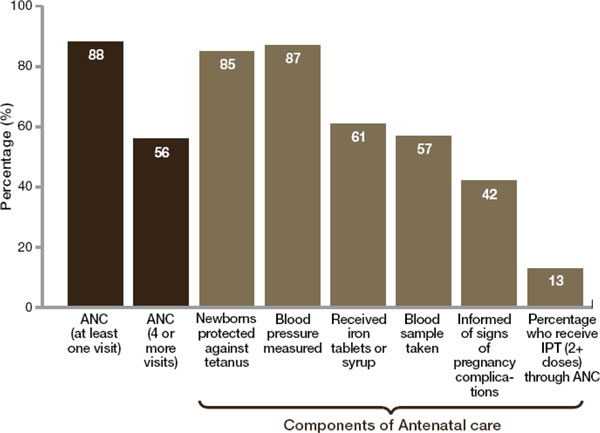
**Median coverage of antenatal care services, Countdown to 2015 priority countries**. Source: Born Too Soon Report [1]. Data source [1]: UNICEF Global Databases, February 2012, based on Demographic Health Surveys, Multiple Indicator Cluster Surveys and other national surveys. Note: Countdown to 2015 priority countries with available data, 2006-2010. Acronyms used: ANC = antenatal care; IPT = intermittent presumptive treatment.

Figure 2 shows median coverage of antenatal care services to pregnant women living in the Countdown to 2015 priority countries where more than 95% of all maternal and child deaths occur [58]. Consistent with the global data, the figure shows high levels of at least one visit of antenatal care across the Countdown countries, but considerably lower levels of the recommended four or more antenatal care visits. This indicates that the majority of women in these countries are not benefiting from the recommended basic package of antenatal care services. The figure also highlights substantial gaps in the quality of care pregnant women are receiving during antenatal care visits, with coverage levels of specific evidence-based interventions considerably lower than the ideal of universal coverage. A similar review of gaps in the provision of key components of antenatal care in sub-Saharan Africa, where the rates and absolute numbers of preterm birth are among the highest in the world, similarly showed low levels of coverage of several recommended components [59,60]. These missed opportunities are a call to action for strengthening health systems in the Countdown priority and other low- and middle-income countries including ensuring practitioners are equipped with the skills and necessary drugs and equipment to deliver effective antenatal care that can potentially reduce the risk of preterm birth and improve health outcomes of the premature baby.

Table 2 shows coverage levels of preterm birth-related interventions available from a World Health Organization multi-country study on the prevalence of maternal near- miss cases with a key objective of mapping the use of evidence-based interventions during childbirth in health care facilities [61]. At the time of publication, country-specific data were available only for the Latin American countries involved in the study, and Mexico is presented as an example. The low coverage levels shown in Table 2 should serve as a startingpoint for dialogue with governments, development partners and health care providers in the study countries on developing strategies for increasing the uptake of these science-based interventions.

**Table 2:** Coverage levels of key interventions, pregnant women in labour, 24 to 34 weeks, after 3 hours in the hospital, World Health Organization 18 country study

Intervention	All countries*	Mexico
Antenatal corticosteroids	56.4%	53.8%
Tocolytic agents		
Beta mimetics	15.8%	8.2%
Calcium channel blockers	9.9%	7.7%
Magnesium sulphate	7.8%	4.9%
Oxytocin antagonists	1.2%	2.4%

*Countries include Afghanistan, Argentina, Cambodia, China, Ecuador, Japan, Kenya, Mexico, Mongolia, Nepal, Nicaragua, Pakistan, Paraguay, Peru, Philippines, Thailand, Sri Lanka and Vietnam.

Source: Born Too Soon report [1]. Data source: Souza et al., 2011. Preliminary results, World Health Organization, Multi-country survey on maternal and newborn health.

### Priority research themes for the pregnancy period and childbirth

**Figure 3:**
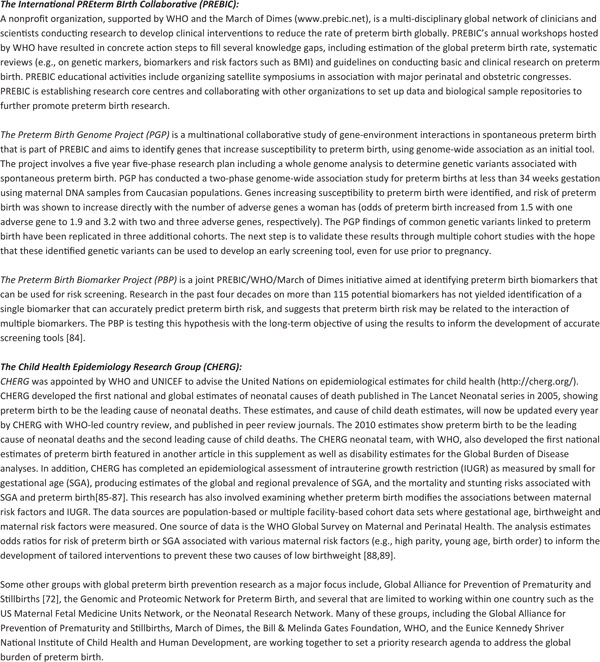
**Building the evidence base on preterm birth**.

There is an imperative need for research on preterm birth that can yield quality data on the efficacy of existing and promising interventions delivered individually or as a package during the pregnancy period. There is an equally critical need for implementation research to improve the scale up of proven policy and health care interventions in low- and middle-income countries. There has been increasing interest in preterm birth in recent years and in developing a comprehensive research paradigm to address this growing cause of neonatal mortality. A major emphasis of the paradigm has been on discovery science to shed light on the basic biology of normal and abnormal pregnancies so that the pathological process of preterm birth can be understood and effective interventions developed. This research will also potentially result in the development of better screening tools for the prediction and prevention of preterm labour. The link between genetics, gene-environment interactions and preterm birth is being investigated with the hope of gaining an understanding of differences in preterm birth rates across racial and ethnic groups that can be translated into more effective and equitable clinical care guidelines including counselling and screening services [26,62]. Research is underway to explore the connections between maternal infections, nutritional status and preterm birth within a broader framework of research on prematurity, low birthweight and intrauterine growth restriction (Figure 3). In addition, research on the social determinants of preterm birth is being conducted to explore the pathways between social disadvantage, behavioural and lifestyle factors and preterm birth [63-65]. A major goal of this research is to help develop strategies for addressing social and economic disparities in preterm birth rates, inequities in access to needed care between and within countries as well as how to best integrate social and behavioural interventions into antenatal care.

Figure 3 presents information on select ongoing research activities to generate the evidence needed to inform the development of effective preventive programmes and policies. Table 3 summarizes the key interrelated areas of priority research on preterm birth in the pregnancy period, emphasizing that simultaneous progress in each of these areas is critical for achieving reductions in preterm birth rates and optimal birth outcomes for the premature baby.

**Table 3:** Research priorities during pregnancy to reduce preterm birth rates and to benefit the preterm baby

**Description**
Epidemiological research to:
• Examine the relationships between maternal risk factors and preterm birth at a population level (e.g., nutritional, infection, age and other socio-demographic factors)
**Discovery**
Basic science research on normal and abnormal pregnancies to:
• Identify the causal pathways leading to preterm labour and birth
• Understand the gestational clock triggering the onset of labour
• Explore the genetic determinants of preterm birth and genetic-environment interactions increasing risk of preterm birth
**Development**
Translational research to:
• Develop simple screening tools based on the findings of biological and genetic research for identifying women at high risk of preterm birth and preterm labour
• Develop robust diagnostic tools for universal application (e.g., anaemia, syphilis)
**Delivery**
Clinical trials and other studies to:
• Build the evidence base on available and promising interventions
• Determine effectiveness of interventions delivered individually and as packages of care
Implementation research to:
• Address coverage gaps by increasing the availability of antenatal care and women's ability to access services around the world
• Address quality of care gaps by increasing the uptake of evidence-based interventions and intervention packages by health care providers (e.g., syphilis testing and screening, blood pressure monitoring during antenatal care visits, etc.)

Source: Born Too Soon report [1].

### Prescription for action during the pregnancy period and childbirth

A standard approach to preterm birth prevention during the pregnancy period hinges upon the findings of the ongoing research on the causal pathways to preterm delivery. Clinical management practices will need to remain flexible as practitioners decide upon a course of action based on the particular characteristics and set of risk factors of their individual women clients. There are, however, basic steps that countries around the world can introduce now and as resources permit to begin making strides towards addressing this growing public health problem. These steps are summarized in Table 4 and described in more detail below.

**Table 4:** Actions during pregnancy to prevent or manage preterm birth

**Invest and plan**
• Ensure national policies and guidelines exist and provide adequate protection of pregnant women and universal access to comprehensive antenatal, labour and birth, emergency obstetric and postnatal care.
• Allocate adequate resources for the provision of equitable and high-quality antenatal care, and removal of barriers to care such as user fees.
**Implement**
• Seize opportunities to leverage resources, approaches, and training opportunities from existing programmes (including non-health programmes)
• Ensure the existence of a functional referral system, procurement system, an adequately trained and supervised health work force, and quality services for all pregnant women. Inform communities about the importance of antenatal, childbirth and postnatal care for all women, and warning signs including early recognition of preterm labour.
**Inform and improve programme coverage and quality**
• Address data gaps and increase sound monitoring and evaluation of programmes to improve service quality and outreach to the poorest populations.
• Prioritize implementation research to promote the scale up of effective interventions in different contexts and across different population groups.
**Innovate and undertake implementation research**
• Invest in discovery research on the basic biology of normal and abnormal pregnancy, genetic determinants of preterm birth, and epidemiological research on maternal risk factors to provide the evidence base needed for the development of effective prevention and treatment strategies.
We all share in the responsibility of making sure pregnant women around the world receive the care they need for healthy birth outcomes.

Source: Born Too Soon report [1].

As a first step, national policies and guidelines for comprehensive antenatal, labour and delivery, emergency obstetric and postnatal care should be established in all countries to promote universal access to quality maternal and perinatal services. Pro-poor legislation (e.g., to abolish user fees, introduce conditional cash transfer programmes, etc.) for maternal and perinatal services should be considered in low- and middle-income countries where out-of-pocket expenses are high and/or coverage levels of antenatal and delivery care interventions are low. Protective legislation is needed to improve general working conditions for pregnant women, and to reduce pregnant women's exposure to potentially harmful environmental, behavioural and lifestyle risk factors such as second-hand smoke and violence against women.

The implementation of national and professional policies and guidelines that are in-line with science-based recommendations, such as those made by WHO, needs to be prioritized. All countries and their partners should allocate sufficient resources to strengthening health care systems to facilitate the implementation process and enable the equitable and early delivery of quality antenatal care. Resources also are needed to develop clearly defined care and referral pathways. Prevention needs to be prioritized through improving access to screening and diagnostic tests and appropriate treatment during antenatal care with adequate follow-up and referral of women identified at high risk of preterm birth.

Efforts to strengthen health systems must include increased and regular training opportunities for health care providers on the use of effective interventions, and on tailoring clinical care to the individual woman based on her risk profile throughout the duration of her pregnancy. The delivery of all recommended components of antenatal care should be regularly instituted and monitored through supportive supervision.

These health care system readiness efforts need to be complemented by communication and education campaigns to increase demand for maternal and perinatal care services and to ensure prospective parents and community members are fully informed about a healthy pregnancy and potential complications.

In settings with greater capacity, professional policies regulating assisted reproductive technologies and infertility treatments should be put into place to reduce the number of multiple gestations at higher risk of preterm birth. Genetic counselling and adequate counselling for women over the age of 35 on pregnancy risks also should be included as components of antenatal care.

All women should be provided with access to quality antenatal care services and a functional referral system throughout pregnancy. Women should be encouraged to give birth in healthcare facilities and have access to health care facilities where quality maternal services are guaranteed. Community members need to be informed about the importance of all women receiving antenatal care and delivering in functioning health care facilities as well as warning signs in pregnancy including preterm labour. In low- and middle-income countries, communities need to be supported in developing appropriate mechanisms that enable women to seek care, particularly the most disadvantaged women who face multiple barriers to accessing timely care.

The integration of effective services should be promoted to best use antenatal care visits as a platform for the delivery of a package of interventions and to leverage scarce resources for women's and children's health. The integration of HIV, malaria, and social and financial support programmes into routine antenatal care, for example, represents an efficient use of resources with potential to reach a large majority of pregnant women.

Health care facilities need to be equipped with trained staff and ample and consistent supplies of drugs and equipment so that women can be guaranteed provision of antibiotics for pPROM, antenatal corticosteroids, progesterone, magnesium sulphate and tocolytics as needed.

Countries need to engage in collaborative and multi-center research for the development of high-quality evidence on available and promising interventions delivered singly and as a package during antenatal care or labour and delivery. Adequate funding is needed for basic science research on the etiology of preterm birth, the physiological processes of healthy and abnormal pregnancies and the linkages between preterm birth and genetics, environment, stress and depression, infection and nutrition. Findings from such research will guide the development of preventive interventions, diagnostic markers and related screening tools and better clinical management guidelines for all women and for women at high risk of preterm birth. These cross-country, multi-disciplinary research efforts will ultimately facilitate the discovery and development of interventions with universal application.

Implementation research is essential for determining how research findings can be best translated into practical application in low- and middle-income countries where resource constraints and inequities in intervention coverage are more pronounced and where innovations in service delivery strategies are most needed. Efforts are needed, for example, on how to most effectively integrate evidence-based interventions into antenatal care in specific contexts so that pregnant women are reached with comprehensive and culturally appropriate packages of care.

National audit and other monitoring and evaluation systems at facility level need to be put into place as a quality control measure to track preterm birth rates and the use of effective services during antenatal care and labour and delivery.

In all settings, ongoing monitoring and evaluation of the implementation of guidelines and policies are essential for ensuring that pregnant women receive high-quality care based on the best evidence.

## Conclusion

The pregnancy period is a time of great excitement for prospective parents. It represents a time period when a woman can be reached through a variety of mechanisms with interventions aimed at reducing her risk of a preterm birth and improving her health and the health of her unborn baby. One key mechanism is routine antenatal care during which a woman should receive a range of effective services at multiple times throughout her pregnancy that are tailored to her individual risk profile. She also can benefit from supportive policies and programmes that increase her access to quality care and protect her from potentially harmful exposures. The message is clear: We all need to work together to make it possible for women everywhere to receive the care they need during pregnancy, labour and delivery as a starting point for successfully addressing the growing problem of preterm birth.

This involves simultaneous and coordinated action in the three main areas of service delivery, discovery and development. Although there are substantial gaps in our knowledge of the underlying causal pathways of preterm labour and delivery, we know there are proven interventions that need to be scaled up now. Governments and their partners must prioritize the equitable delivery of these interventions through innovative and cost-effective strategies. At the same time, basic research and discovery science must be promoted to build the evidence base so that effective preventive and treatment interventions can be designed. This will require collaborative partnerships across institutions and countries. As the evidence is generated, resources need to be allocated to its translation into new and better screening and diagnostic tools and other interventions aimed at saving maternal and newborn lives. The time is now to put this action plan into motion.

## Abbreviations

WHO: World Health Organization; IUGR: Intrauterine growth retardation; SGA: Small for gestational age; IPTp: Intermittent Presumptive Treatment during pregnancy for malaria; PREBIC: PREterm Birth Collaborative; PGP: Preterm Birth Genome Project; PBP: Preterm Birth Biomarker Project; CHERG: The Child Health Epidemiology Research group.

## Conflict of interest

The authors declare that they have no competing interests.

## Authors' contributions

JR drafted the paper, and all authors reviewed and contributed.

## Supplementary Material

Additional file 1 **In line with the journal's open peer review policy, copies of the reviewer reports are included as **additional file 1. Click here for file
