# Supplementary material for: Born Too Soon: Care during pregnancy and childbirth to reduce preterm deliveries and improve health outcomes of the preterm baby
Source: Reprod Health. 2013 Nov 25;10(Suppl 1):S4. doi: 10.1186/1742-4755-10-S1-S4 (PMC3842748; doi:10.1186/1742-4755-10-S1-S4)
Supplement: Additional file 1 — In line with the journal's open peer review policy, copies of the reviewer reports are included as additional file 1. [file 1742-4755-10-S1-S4-S1.doc]

**Referee’s comments to the authors– this sheet WILL be seen by the author(s) and published with the article**

| Title | **Care during pregnancy and childbirth** |
| --- | --- |
| Author(s) | Jennifer Requejo, Fernando Althabe, Mario Merialdi, Matthais Keller, Joanne Katz and Ramkumar Menon |
| Referee’s name | Jeffrey Smith |

**When assessing the work, please consider the following points, where applicable:**

1. Is the question posed by the authors new and well defined?
2. Are the methods appropriate and well described, and are sufficient details provided to replicate the work?
3. Are the data sound and well controlled?
4. Does the manuscript adhere to the relevant standards for reporting and data deposition?
5. Are the discussion and conclusions well balanced and adequately supported by the data?
6. Do the title and abstract accurately convey what has been found?
7. Is the writing acceptable?

Please make your report as constructive and detailed as possible in your comments so that authors have the opportunity to overcome any serious deficiencies that you find and please also divide your comments into the following categories:

- Major Compulsory Revisions (which the author must respond to before a decision on publication can be reached)
- Minor Essential Revisions (such as missing labels on figures, or the wrong use of a term, which the author can be trusted to correct)
- Discretionary Revisions (which are recommendations for improvement but which the author can choose to ignore)

Where possible please supply references to substantiate your comments.

When referring to the manuscript please provide specific page and paragraph citations where appropriate.

**General comments:**

The style of writing is a bit opaque. For example, the sentence *“Systematic reviews and meta-analyses show that antibiotic treatment for infection and periodontal care does not reliably reduce the risk of preterm birth, and effectiveness may depend upon when during pregnancy the infection or periodontal disease is detected and treated.”* This is very hard to take any guidance from. What should a provider or health manager do with this information?

They paper takes an interesting approach to stating the recommendations in that it says a practice has a positive influence, but it doesn’t give much detail about the practice. In another sentence: *Administration of progesterone to prolong pregnancy in high-risk women with a history of previous preterm birth has been shown effective in preventing a recurrence of preterm birth in these women and in decreasing the prevalence of low birthweight.* This, and other sections of the paper seems to intentionally leave out the specifics. It seems that the intentions of the authors are to present information on interventions, but not present the specifics of any of those interventions. This substantially limits the usefulness of this paper.

*(continue on the next sheet)*

*Continued*:

**Major compulsory revisions:**

Statements need to be more completely referenced. The entire section under ***Management of women in preterm labor to improve survival chances of the premature baby*** has no references (except for what is in the box on antenatal corticosteroids, but makes important definitive statements.

**Minor essential revisions:**

Discussion of PPROM management is very brief.

There is no mention of group antenatal care, which has also been shown to reduce PTB rates, in selected populations in the US.

**Discretionary revisions:**

Certain WHO documents refer to PPROM as preterm prelabor rupture of membranes.

**Referee’s comments to the authors– this sheet WILL be seen by the author(s) and published with the article**

| Title | **Care during pregnancy and childbirth** |
| --- | --- |
| Author(s) | Jennifer Requejo, Fernando Althabe, Mario Merialdi, Matthais Keller, Joanne Katz and Ramkumar Menon |
| Referee’s name | Julia Hussein |

**When assessing the work, please consider the following points, where applicable:**

1. Is the question posed by the authors new and well defined?
2. Are the methods appropriate and well described, and are sufficient details provided to replicate the work?
3. Are the data sound and well controlled?
4. Does the manuscript adhere to the relevant standards for reporting and data deposition?
5. Are the discussion and conclusions well balanced and adequately supported by the data?
6. Do the title and abstract accurately convey what has been found?
7. Is the writing acceptable?

Please make your report as constructive and detailed as possible in your comments so that authors have the opportunity to overcome any serious deficiencies that you find and please also divide your comments into the following categories:

- Major Compulsory Revisions (which the author must respond to before a decision on publication can be reached)
- Minor Essential Revisions (such as missing labels on figures, or the wrong use of a term, which the author can be trusted to correct)
- Discretionary Revisions (which are recommendations for improvement but which the author can choose to ignore)

Where possible please supply references to substantiate your comments.

When referring to the manuscript please provide specific page and paragraph citations where appropriate.

**General comments: This paper is a review of current strategies to reduce premature births, the gaps in knowledge and research needs. It fulfils a need for a summary paper on the topic. The paper is well written and comprehensive, but could do with some minor improvements.**

**Minor essential revisions:**

1. The method used in compiling the references used in the paper is not explained.
2. There is an explanation about the source of the paper in the acknowledgements section. I think this is too late, and too hidden. Perhaps a brief explanation of the context of the Born too Soon report could be provided within the manuscript itself at the beginning.
3. The introduction to the paper is rather abrupt. It would be helpful for the reader if a framework is provided for the subsequent topics discussed in a diagram or flow chart of some sort as an introduction.
4. Under the description of ‘priority packages’ (page 2), the explanation of medical and non medical indications is rather confusing. It would be useful to differentiate medical (e.g. diabetes) conditions from obstetric ones (e.g. pre-eclampsia).
5. The interventions required for medical complications in pregnancy (diabetes, thyroid disease) are different from those for

**Discretionary revisions:**

*(continue on the next sheet)*

*Continued*: different from those of obstetric complications (antepartum haemorrhage, pre-eclampsia). The article would benefit from a little more discussion about these issues and the interventions associated with them. What priority packages have been identified for medical complications in Table 1? Diabetes is mentioned in the text, but surely screening and early detection ought to be part of an antenatal service? Is this a ‘situational intervention? (can’t say I understand this term) ….but the examples given are only on communicable diseases. The examples given under ‘additional interventions’ are also a strange mix of socio-economic and nutritional interventions. Perhaps clearer categorization may help here. There could also be additional examples of non obstetric, medical interventions given in the management of pregnant women at risk?

6. Figure 1 is confusing. The data presented are the proportions of populations reached with interventions, yet the title is about ‘missed opportunities’ – surely the missed opportunities are the population not reached?

## Comments from the Editor:

For all the articles, these should be seen as independent papers that can be read without the need to read the other papers in the series.
